# Two cases of deliberate implant mismatch in knee arthroplasty

**DOI:** 10.1051/sicotj/2020016

**Published:** 2020-06-17

**Authors:** Laura Marie-Hardy, Padhraig O’Loughlin, Michel Bonnin, Tarik Ait Si Selmi

**Affiliations:** 1 Service de Chirurgie Orthopédique, Hôpital de la Pitié-Salpétrière, Sorbonne Université 47, Bd de l’Hôpital 75013 Paris France; 2 Department of Orthopaedic Surgery, Cork University Hospital Wilton T12 DFK4 Cork Ireland; 3 Service de Chirurgie Orthopédique, Clinique Mermoz-Paul Santy 24 Avenue Paul Santy 69008 Lyon France

**Keywords:** Total knee arthroplasty, Unicondylar knee arthroplasty, Mismatch

## Abstract

*Cases*: Knee arthroplasty is increasingly common with good clinical results. However, there is a cohort of patients whose native knee anatomy may not marry well with standard implants. The current authors describe two cases (one unicompartmental knee arthroplasty (UKA), one total knee arthroplasty (TKA)), during which deliberately implanting an implant designed for the contra-lateral distal femur (TKA) or contralateral femoral condyle (UKA) respectively, led to a better fit than correct-sided implants. *Conclusion*: The authors share their experience to raise awareness of a potential solution to such an intra-operative challenge and suggest that implant customisation may ultimately address challenges with grossly abnormal native anatomy.

## Introduction

Joint arthroplasty involves replacing arthritic native articular surfaces with prosthetic alternatives. To optimise fit and function, different sizes of implants are available and implants designed for use in left or right-sided joints e.g. total knee arthroplasty (TKA), or medial and lateral femoral condyles (in the context of unicompartmental knee arthroplasty (UKA)). Despite this, there are situations where a patient’s anatomy does not conform to any available implants. In fact, that might explain disappointing outcomes in some cases. Beard et al. describe an 82% survival rate at 12 years, but with 41% of patients reporting modest or severe pain [1].

The current authors describe two cases of young patients with severe osteoarthritis and grossly abnormal distal femoral geometry which did not facilitate the use of standard correct-sided (left/right in TKA, medial/lateral condyle in UKA) implants.

## Case no. 1

A 56-year-old male patient with Nail-Patella syndrome, presented with bilateral painful, stiff knees. Medial condylar epiphysiodesis had been performed on the left knee, at age 12.

For several years, he had experienced a sense of instability in this joint. The pain was primarily mechanical and was impacting negatively on his occupation as a chemistry teacher.

On examination, both patellae were chronically dislocated with an associated bilateral, partially reducible, genu valgum of 9° and a fixed flexion deformity, with a range of motion of 20°–130°.

**Figure 1:**
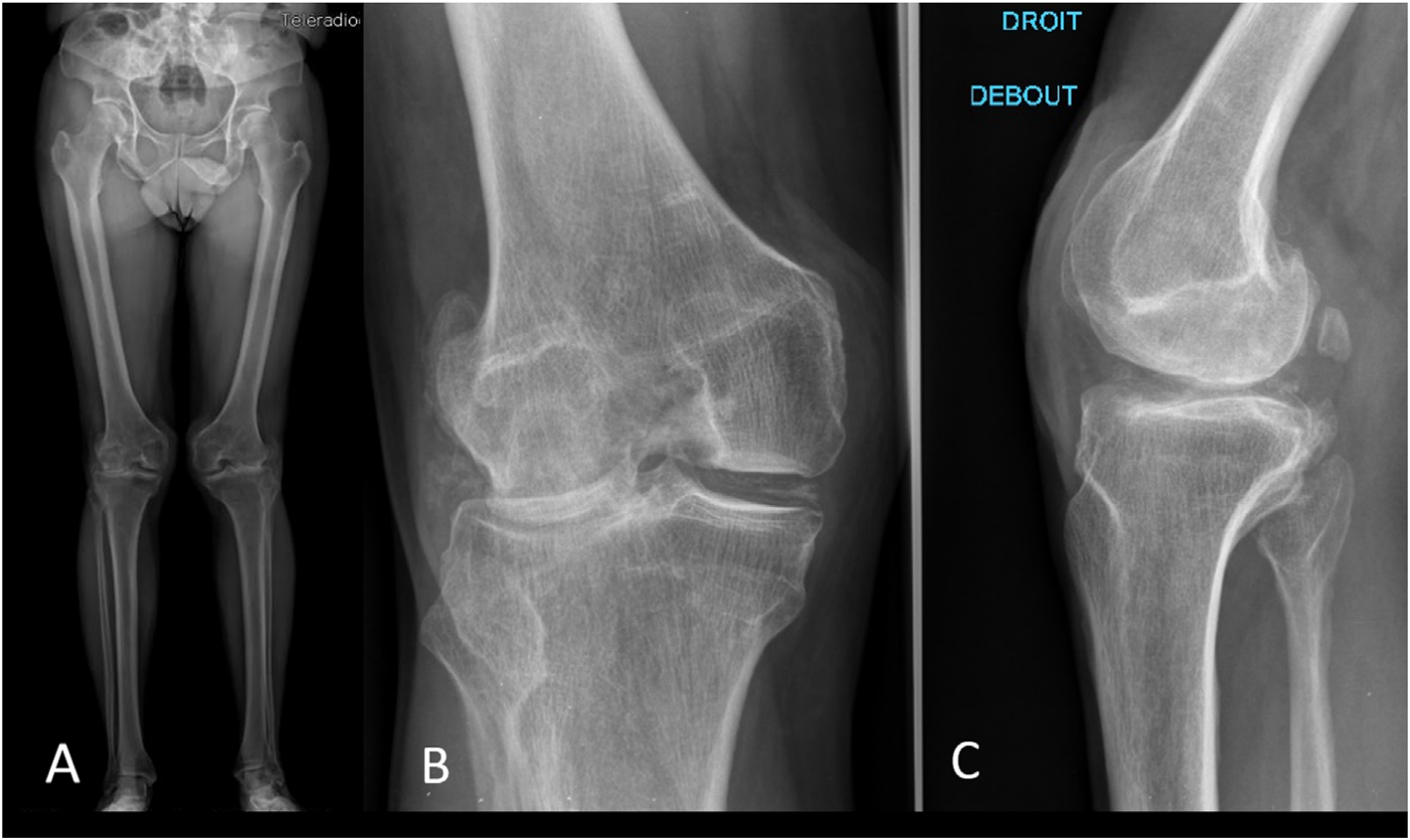
Pre-operative plain radiographs: (A) standing both lower limbs, (B) anteroposterior (AP), (C) lateral view, right knee.

Plain knee radiographs and magnetic resonance imaging (MRI) revealed severe arthritis of both lateral compartments and hypoplasia of the dislocated patellae (Figure 1). The radiographic appearances were quite typical of Nail–Patella syndrome [2].

A range of surgical options were discussed with the patient.

The tibial osteotomy would not have been sufficient to address the fixed flexion deformity, which was one of the patient’s main complaints.

With regard to extensor mechanism re-alignment, the anticipated likelihood of morbidity was relatively high given that the patient had undergone prior surgery to this knee. Moreover, the probability of having to use a constrained prosthesis was also high, with no guarantee of success at addressing the chronic patellar dislocation [3]. Using revision implants with the need of a tibial osteotomy for the exposure would certainly have endangered the integrity of the extensor apparatus and certainly led to a poor functional outcome.

Consequently, lateral unicondylar knee arthroplasty was chosen for the right knee.

The right knee had a range of motion of 10°–130°. The inability to extend fully was less bothersome for the patient on this side but the dislocated patella did cause pain and discomfort. MRI demonstrated intact cruciate ligaments.

**Figure 2:**
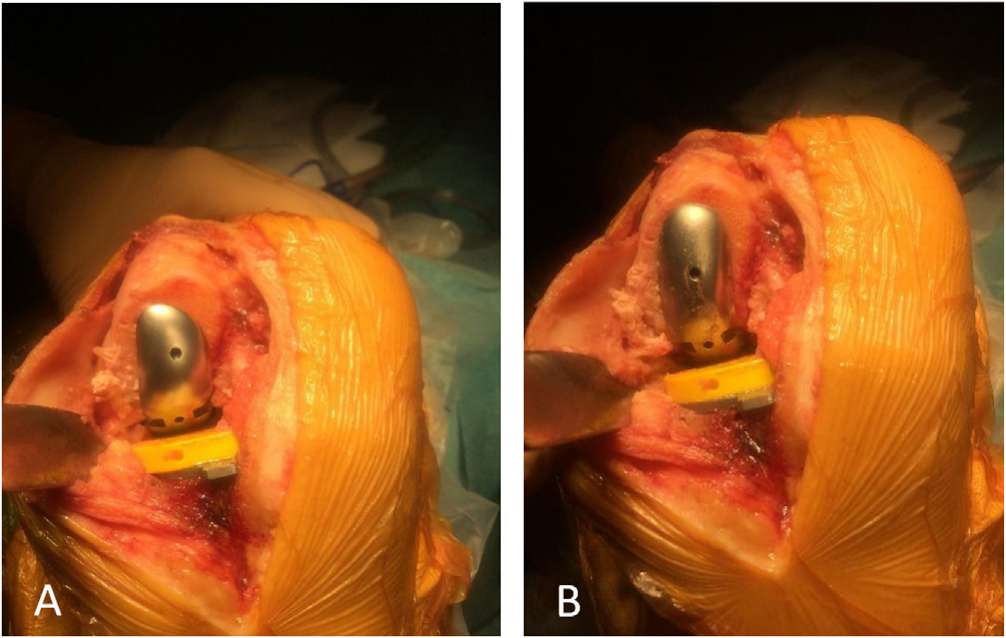
Intra-operative clinical photographs, right knee: (A) anterior view, lateral condyle implant *in situ* (on lateral condyle), (B) anterior view, “medial condyle implant *in situ* (on lateral condyle)”.

Intra-operatively, the lateral femoral implant did not conform to the morphology of the patient’s lateral femoral condyle given the gross deformity present (Figure 2A). Bone coverage was poor, especially on the lateral side with unsatisfactory congruence. The global axis was deflected into a varus deformity.

Subsequently, a trial with a medial femoral condylar implant was significantly more congruent. The implant sat well in the middle of the lateral femoral condyle, leading to appropriate bone coverage and an acceptable mechanical axis (Figure 2B).

Dynamic testing demonstrated adequate patellar tracking with no laxity either in extension or in flexion. Femoral and tibial components were slightly convergent, as required.

Thus, an implant designed for medial compartment implantation was selected to address the unique anatomy of this patient’s lateral compartment. The rationale behind this decision was recorded in the operative note and communicated fully to the patient. Intra-operative images with both implants in situ were taken and discussed with the patient.

**Figure 3:**
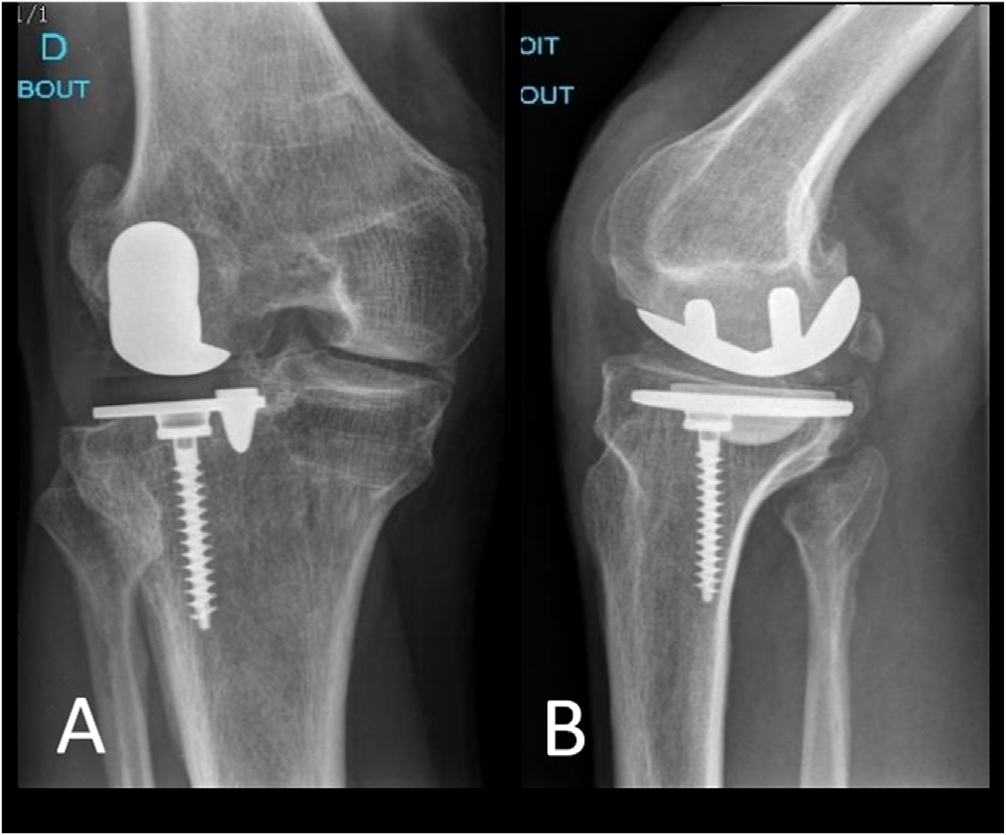
Post-operative plain radiographs, right knee: (A) anterior view, (B) lateral view.

Post-operative radiographs were satisfactory (Figure 3).

At most recent follow-up, at two years post-surgery, the knee has a range of motion of 5°–0°–120°. The patient’s mobility was improved compared to their pre-op status and he mobilised without any walking aid.

The patient was very satisfied with the outcome, with no pain or instability.

## Case no. 2

**Figure 4:**
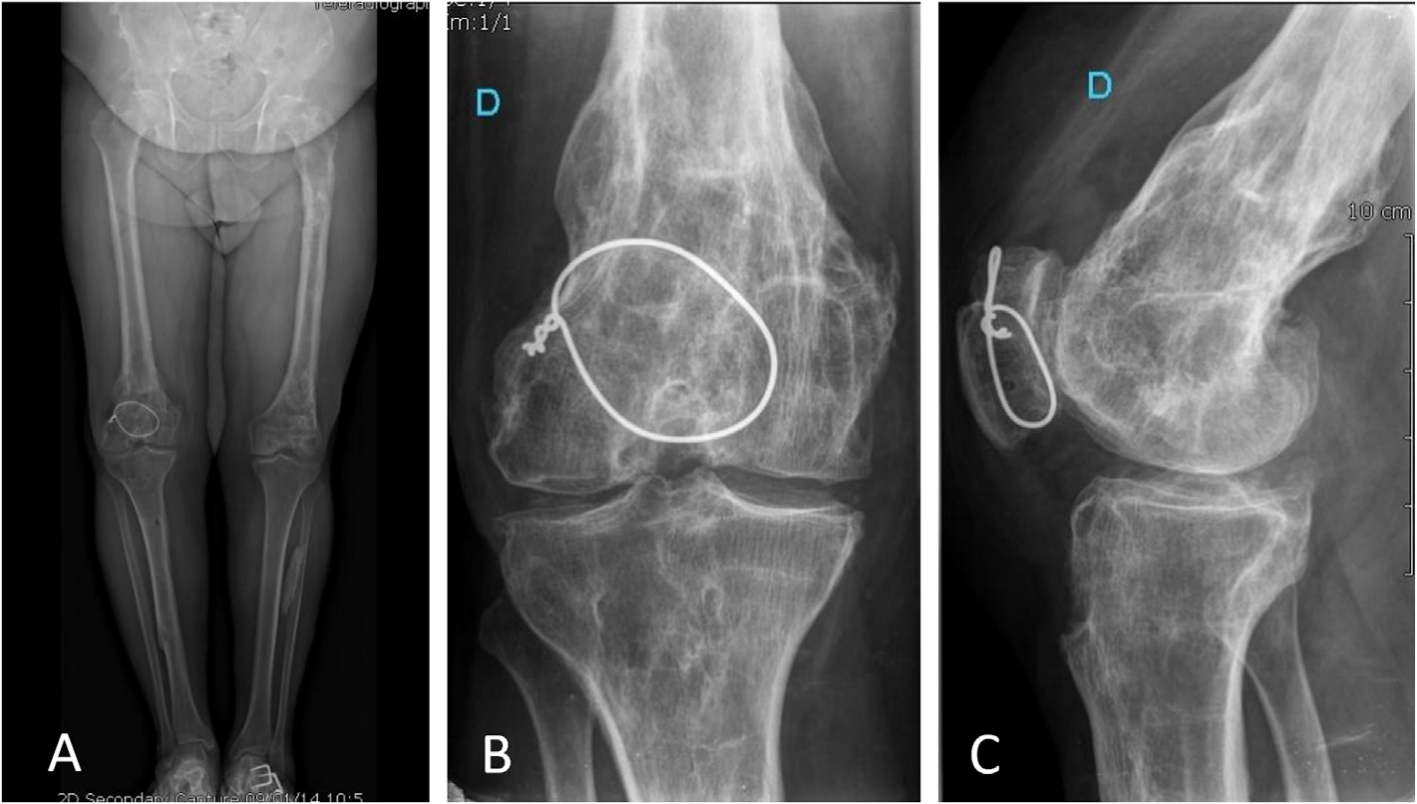
Pre-operative plain radiographs: (A) standing bilateral, (B) AP view, right knee, (C) lateral view, right knee.

A 46-year-old male patient presented with right knee pain and stiffness which was impacting significantly on his daily activities, making it difficult to walk more than 500 m and rendering him unemployed. He had sustained trauma to both lower limbs several years earlier with fractures of both tibiae, his left femoral diaphysis, distal right femur and right patella (which was treated six years prior to his first presentation to the current authors’ institution, with cerclage wiring) (Figure 4).

Clinically there was 15° of genu varus on the right side, which was not reducible. His range of motion was 0°–0°–120°. Plain radiography demonstrated severe tricompartmental post-traumatic arthritis. Additionally, the right distal femur had a recurvatum deformity with lateral translation of the distal fracture fragment and there was evidence of patella baja (Figure 4).

In this case, there were not many surgical options to discuss. The knee arthritis was too severe and global to think about osteotomy or unicondylar arthroplasty and TKR was deemed to be the most reasonable option, despite the patient’s age [4].

**Figure 5:**
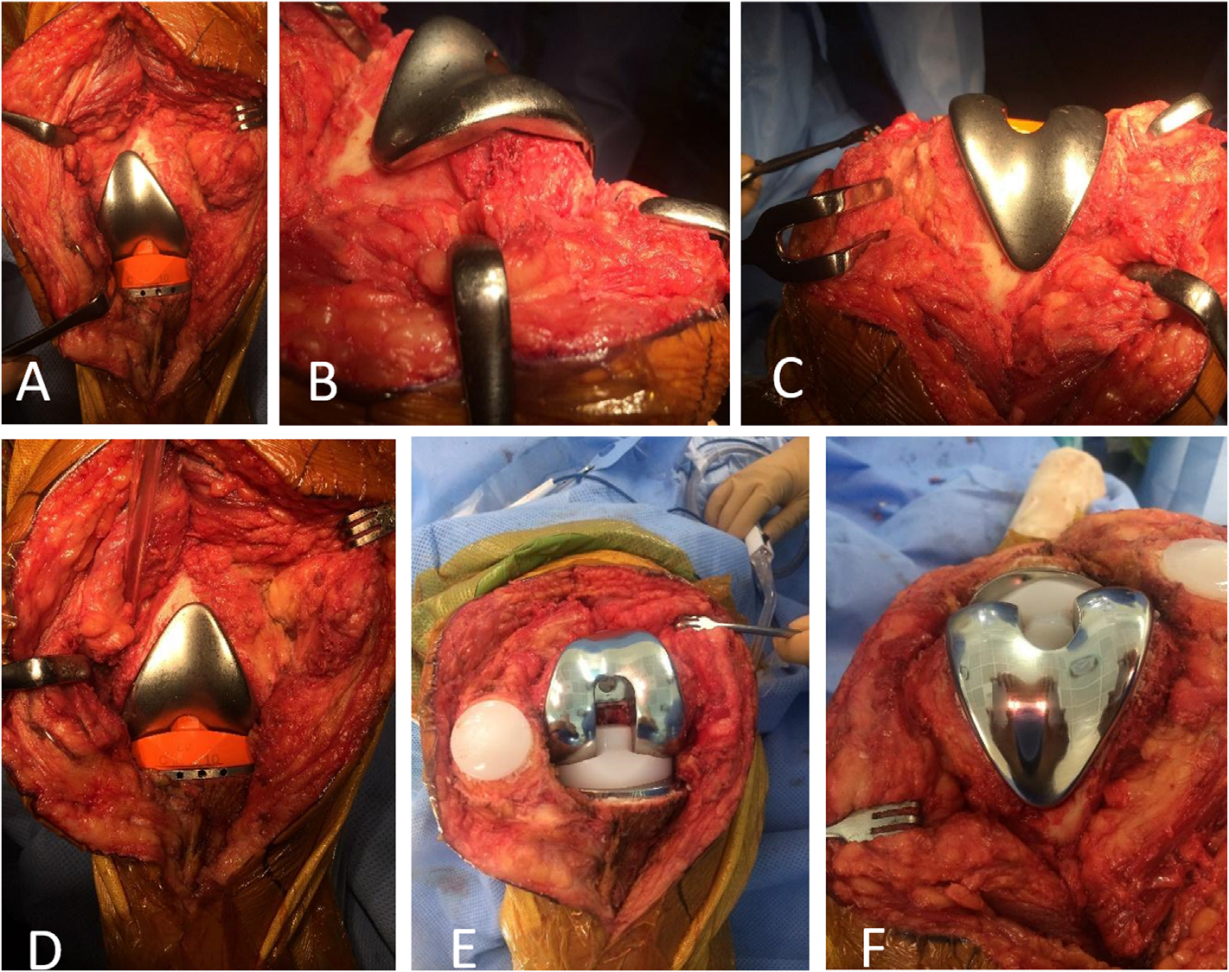
Intra-operative clinical photographs, right knee with right-sided femoral implant *in situ*: (A) anterior view (knee in extension), (B) lateral view (knee in flexion), (C) view from above (knee in flexion) and “right knee with LEFT-sided femoral implant *in situ*”: (D) anterior view (knee in extension), (E) anterior view (knee in flexion), (F) view from above (knee in flexion).

Intra-operatively, the right femoral implant trial was grossly mismatched relative to the distal femur due to the underlying post-traumatic deformity and lateral translation of the native distal femur due to fracture mal-union (Figures 5A–5C). There was medial condylar under-coverage, protuberance of the implant laterally and it was anticipated that this would lead to both functional and soft-tissue problems for the patient.

Thus, it was decided to trial a left-sided femoral component and the result was dramatically more satisfactory, such that this implant, designed for the contralateral side was implanted (Figures 5D–5F).

**Figure 6:**
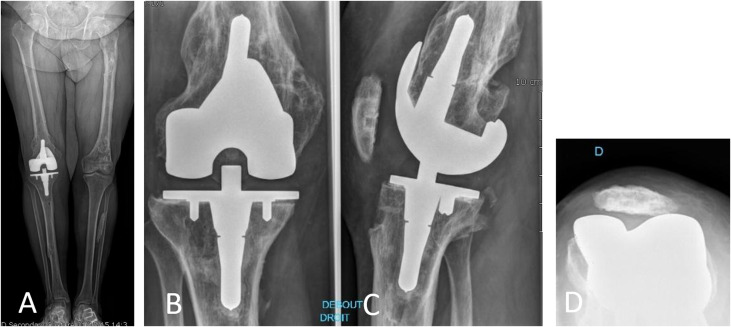
Post-operative plain radiographs: (A) standing both lower limbs, (B) AP, right knee, (C) lateral view, right knee, (D) merchant view, right knee.

At the most recent follow-up, at one-year post-op, the patient reported almost no pain, was able to comfortably negotiate stairs and walk several miles. Mobility was 0–0–125. Radiographs were satisfactory (Figure 6).

## Discussion

The cases detailed above describe the intentional use of implants that were not designed for the specific condyle or specific distal femur to which they were ultimately applied. In the first case, a medial component is applied to the lateral femoral condyle during unicompartmental knee arthroplasty and in the second case, a left femoral component was applied to the right femur.

Choosing to proceed with this mode of component implantation is not straight-forward. There can be legal repercussions if the long-term outcome is sub-optimal. Equally, issues of patient consent come into play, especially if the decision to proceed with the “wrong” implant is an intra-operative one.

There are also situations where no conventional implant, be it for the correct side or not, will match the patient’s anatomy satisfactorily and this leads to the subject of patient-specific solutions. Of course, patient-specific implants have traditionally entailed additional costs as well additional imaging with its inherent cost and exposure to additional radiation (in the case of CT).

Submitting a patient to a second anaesthetic, in order to discuss the choice to proceed with atypical use of an implant along with the inherent risks of temporary wound closure and a second surgery, is not an insignificant action. It is understandable that in these litigious times that one would tend to practice surgery defensively, but it is prudent to do so in a case-by-case manner.

In performing complex arthroplasty surgery, especially those involving anatomical abnormalities e.g. congenital or post-traumatic, it may be worth considering a “mismatch” component, if one is struggling to fit a correctly-sided implant. This option may achieve one’s objectives without the need to resort to patient-specific implants.

However, in the future as bespoke solutions become more ubiquitous and feasible, it is likely that they will become a more elegant and defensible option. 3D-printing may contribute in a positive way to the process also.

Of course, the over-arching theme that the authors seek to convey is that whilst one may be able to serendipitously utilise available implants albeit designed for another side of the bone or the body, greater customisation is needed to avoid the need to resort to such strategies.
